# Retraction Note: microRNA-15b-5p encapsulated by M2 macrophage-derived extracellular vesicles promotes gastric cancer metastasis by targeting BRMS1 and suppressing DAPK1 transcription

**DOI:** 10.1186/s13046-022-02426-x

**Published:** 2022-06-29

**Authors:** Yi Cao, Yi Tu, Jianbo Xiong, Shengxing Tan, Lianghua Luo, Ahao Wu, Xufeng Shu, Zhigang Jie, Zhengrong Li

**Affiliations:** 1 Department of General Surgery, Jiangxi Province, The First Affiliated Hospital of Nanchang University, No. 17, Yongwai Zheng Road, Nanchang, 330006 People’s Republic of China; 2 Department of Pathology, The First Affiliated Hospital of Nanchang University, Nanchang, 330006 People’s Republic of China

**Retraction Note: J Exp Clin Cancer Res 41, 152 (2022)**

**https://doi.org/10.1186/s13046-022-02356-8**

The authors have retracted this article. After publication, the authors realized there were potential errors with figure labels and the cell lines used in the experiments. Additional checks have identified that:

1. Fig. 1c states that co-localization of CD206 and miR-15b-5p is presented, but only images for miR-15b-5p and DAPI are presented.
2. Fig. 1i blots appear to have deleted backgrounds and an artifact in the Calnexin image.
3. Figs. 1b, 3e and 5c use SGC-7901 cells, which are contaminated with HeLa.
4. Fig. 5i MKN-45 data don’t appear to match Fig. S2c.
5. Fig. 7c AGS BRMS1 data don’t appear to match Fig. S2f.
6. Fig. S2a MKN-45 VIM lanes 1 and 2 have similar artifacts on the right side of the bands.
7. The study did not obtain ethics approval prior to the start of the experiment.

The authors have been able to provide alternative data for Figs. 1i, S2a, c and f to address some of these concerns. However, the cell line and ethics issues could not be resolved.

All authors agree to this retraction.

